# Increased PD-L1 and p16 expression are common in oropharyngeal squamous cell carcinoma

**DOI:** 10.2144/fsoa-2021-0039

**Published:** 2021-10-28

**Authors:** Anna Shestakova, Jana Tarabay, Anton Burtsev, Ifegwu Ibe, Jeffrey Kim, Vishal Chandan, William B Armstrong, Tjoa Tjoson, Beverly Wang

**Affiliations:** 1 Department of Pathology, University of Utah and ARUP laboratories, Salt Lake City, UT 84108, USA; 2 Department of Pathology, University of California, Irvine, CA 92868, USA; 3 Department of Computer Science, University of California, Irvine, CA 92697, USA; 4 Department of Otolaryngology – Head & Neck Surgery, University of California, Irvine, CA 92868, USA

**Keywords:** hypopharynx, larynx, mouth, oropharynx, papillomaviridae, pembrolizumab, radiation, squamous cell carcinoma of head and neck

## Abstract

Overexpression of p16 is closely related to human papillomavirus (HPV)-associated oropharyngeal squamous cell carcinoma (SCC) and pertains a prognostic relevance. Programmed cell death 1-ligand 1 (PD-L1) is another important marker, as anti-PD-L1 immunotherapy is available. Retrospective analysis of 57 cases of the SCC involving oropharynx (27 cases), hypopharynx (5 cases), larynx (11 cases), and oral cavity (14 cases) was performed. Each case was scrutinized for the basaloid morphology, p16, and PD-L1 expression. Basaloid morphology was identified in 47% of total cases. The majority of basaloid SCC variants were located in the oropharynx (89%). High expression of p16 was mostly observed in the oropharynx. High PD-L1 expression was seen predominantly in oropharyngeal and hypopharyngeal locations. Further studies in a larger cohort are necessary to correlate PD-L1 and p16 expression with survival.

## Lay abstract

Squamous cell carcinoma is the most common malignancy in the head and neck area to include oral cavity, oropharynx, hypopharynx, and larynx. Patients' prognosis depends on a variety of factors including anatomic location of the cancer and biomarkers that are associated with it. Programmed cell death 1-ligand 1 (PD-L1) is a powerful biomarker that can be useful to justify immunotherapy with anti-programmed cell death protein 1 (PD-1) (Keytruda). This study performed a retrospective analysis of 57 cases of the squamous cell carcinoma involving the head and neck area with basaloid morphology, p16, and PD-L1 expression. Further studies in a larger cohort are necessary to correlate PD-L1 and p16 expression with survival.

Squamous cell carcinoma of the head and neck (HNSCC) is the sixth most common cancer worldwide with approximately 630,000 new patients diagnosed annually [1]. Over 90% of HNSCC is associated with the mucosa of the oral cavity, oropharynx, hypopharynx, and larynx [1,2]. The oropharynx is comprised of palatine tonsils, base of tongue, soft palate, uvula, and posterior pharyngeal wall. Oropharyngeal squamous cell carcinoma (OPSCC) constitutes 60% of the squamous cell carcinoma of the head and neck [3]. Cigarette smoking, alcohol, and human papillomavirus (HPV) infection contribute primarily to the pathogenesis of oropharyngeal SCC. HPV infection status is also a strong prognostic factor. Over 90% of oropharyngeal squamous cell carcinoma is associated with HPV high-risk type 16 [4]. HPV-associated OPSCC shows a superior three-year survival when compared to conventional HNSCC [5]. Patients with HPV-associated OPSCC demonstrate a superior outcome compared to non-HPV-associated OPSCC [3].

Expression of the tumor suppressor gene, p16, appears to correlate with HPV infection in oropharyngeal SCC [6]. The mechanism of p16 overexpression is thought to be secondary to viral components E7 interfering with the function of Rb, leading to up-regulation of p16. Therefore, immunohistochemical staining of p16 is used commonly as a surrogate marker of HPV status in oropharyngeal squamous cell carcinoma. In contrast, it has been demonstrated that p16 expression is an imperfect surrogate biomarker of HPV infection and is controversial for its prognostic value in non-oropharyngeal HNSCC. It has been reported, that p16 expression did not correlate with HPV positivity in approximately 10% of cases of penile squamous cell carcinoma [7,8]. In addition, overexpression of p16 in in oral cancers was demonstrated to be unrelated to HPV [9,10].

Programmed cell death 1-ligand 1 (PD-L1) is an immune modulatory molecule in cancer cells that inhibits cytotoxic T cells and induces evasion of tumor cells from the immune system [11]. PD-L1 binds to programmed cell death protein 1 (PD-1) in T-cells in the tumor microenvironment to modulate immunity. This is one of the mechanisms by which cancer cells evade the immune system. Numerous studies have identified wide range of levels of PD-L1 expression in HNSCC tissues [12]. Furthermore, HPV-positive OPSCC exhibit a higher expression of PD-L1 than HPV-negative patients with OPSCC [13]. However, in patients with non-OPSCC, the expression of PD-L1 and p16, as well as their association, remains unclear. Furthermore, the prognostic value of PD-L1 in HNSCC has not been clearly established for sites other than the oropharynx.

The current study reports association of HNSCC with basaloid histomorphology, p16, and PD-L1 expression as well as other clinicopathological characteristics.

## Materials and methods

Retrospective analysis of 57 cases of the SCC involving the oropharynx (32 cases) (including tonsil [16 cases] and base of the tongue [11 cases]) and hypopharynx (5 cases); larynx (11 cases) and oral cavity (14 cases) (including oral tongue [6 cases], lip [2 cases], buccal, and alveolus [6 cases]) that were diagnosed at our institution from January 2018 to May 2019 was performed. Each case was scrutinized for presence of basaloid morphology, p16, and PD-L1 expression by immunohistochemistry. PD-L1 expression using a recombinant rabbit monoclonal anti-PD-L1 antibody (SP263, Roche, Ventana, AZ, USA) was scored independently by three pathologists. Combined positive score (CPS) of low/absent (≤1%), intermediate (≥1%) and high (≥10%) was calculated in at least 200 cells to include tumor cells and mononuclear inflammatory cells with any membranous staining. Human placental tissue was used as an appropriate internal staining control demonstrating membranous staining of the syncytotrophoblast layer of the placenta, while showing absence of staining in the stromal and vessels.

### Statistics

Due to the small sample size the Fisher exact test was used to calculate p-values. The null hypothesis was that clinicopathologic characteristics are independent of anatomical site of involvement by head and neck squamous cell carcinoma. R statistical package (version 3.4.4), which was invoked from an open-source programming language Python (version 3.6.9), was used to calculate p-values. p-values < 0.05 were considered significant.

### Patients

Between January 2018 and May 2019, 57 patients with mucosa-associated HNSCC that were diagnosed and/or treated at the University of California, Irvine Medical Center (CA, USA) were retrospectively reviewed. Clinical information regarding patients including patient age, sex, tobacco use, alcohol consumption, and treatment history was collected. Pathologic cancer staging was established according to the 8th American Joint Committee on Cancer Staging [12]. The current study was approved by the Institutional Review Board (IRB) of University of California, Irvine Medical Center (HS-2019-5255). Since the current study was a retrospective study using tissue collected for treatment and/or diagnostic purposes, patient consent was waived as per IRB.

### Immunohistochemical staining & interpretation of PD-L1 and p16

Immunohistochemical (IHC) procedures for p16 and PD-L1 were performed as per manufacturer recommendations (antibodies CINtec and SP263, respectively) on a Ventana Benchmark Ultra instrument (Roche). Briefly, individual tissue sections were obtained from the formalin-fixed paraffin-embedded (FFPE) tissue blocks. Tissue sections were cut at 4 μm thickness and mounted on a positively charged slides. Each section was subjected to the routine hematoxylin and eosin staining, p16, and PD-L1 IHC staining.

#### PD-L1 antibody IHC staining and interpretation

Ventana PD-L1 (SP263, Roche, cat. nhe)o. 740-4907) is a recombinant rabbit monoclonal antibody produced as purified cell culture supernatant. One 5 ml dispenser of Ventana PD-L1 SP263 (Roche) contains approximately 8 μg of antibody, which is sufficient for 50 reactions. After diluting the antibody in a 0.05 M Tris-HCL with 1% carrier protein, specific antibody concentration was approximately 1.61 μg/ml. Subsequently, Ventana PD-L1 SP263 antibody was detected using a heptanated secondary antibody followed by a multimer anti-hapten-HRP conjugate (OptiView DAB IHC Detection Kit, cat. no. 760-700). Human placental tissue was used as a positive and a background control for Ventana PD-L1 SP263. Brown colored DAB reaction product precipitates at the antigen sites localized by the Ventana PD-L1 (SP263) assay. The stained slides were interpreted by three independent pathologists using light microscopy. Tumor cells and mononuclear inflammatory cells exhibiting any membranous staining were defined as positive for PD-L1. PD-L1 expression was defined as a combined positive score (CPS), which is the number of PD-L1 staining cells (tumor cells, lymphocytes, macrophages) divided by the total number of viable tumor cells, multiplied by 100. The distribution of staining was categorized as follows: CPS of low/absent (≤1%), intermediate (1–10%) and high (≥10%). The CPS was calculated in at least 200 cells to include tumor cells and mononuclear inflammatory cells with any membranous staining.

#### p16 antibody IHC staining and interpretation

Ventana CINtec Histology uses a mouse monoclonal anti-p16 antibody clone E6H4 to detect mouse p16^INK4A^ protein in FFPE (CINTtec Histology, Roche, cat. no. 705-4793). One CinTec Histology 5 ml dispenser contains approximately 5 μg of antibody, which is sufficient for 50 reactions. After diluting the antibody in a 0.05 M tris-HCL with 1% carrier protein, specific antibody concentration was approximately 1.0 μg/ml. Subsequently, the specific antibody is localized using brown-colored OptiView DAB IHC detection kit (Ventana, Roche, cat. no. 760-700). Human placental tissue was used as a positive and a background control for Ventana PD-L1 SP263. The stained slides were interpreted by two independent pathologists using light microscopy. Tumor cells exhibiting diffuse, strong, and continuous nuclear and cytoplasmic staining were defined as positive for p16.

## Results

### Clinicopathologic characteristics of patients

The clinical characteristics of the 57 patients included in this study are presented in Table 1. The mean age of the patients at the latest follow-up was 66 years (range: 29–87 years). 46 were men (81%) and 11 were women (19%). The follow-up period ranged from 12 to 48 months. At the latest follow-up, in May 2020, 41 patients were alive, 11 patients succumbed to the disease, and 6 patients were lost to follow up. Smoking and excessive alcohol consumption was noted by 37 (65%) and 5 (9%) patients, respectively. The anatomical sites of involvement were classified as oropharynx (to include tonsil and base of the tongue) 27 patients (47%), oral cavity 15 patients (26%), hypopharynx 4 patients (7%), and larynx 11 patients (19%). Out of 57 patients, 33 were staged according to the AJCC (American Joint Committee on Cancer) 8^th^ edition as follows: 20 patients (35%) were diagnosed as having stage I/II disease and 13 patients (23%) had stage III/IV disease. Twenty-four patients (42%) were not staged due to the biopsy nature of the surgical specimen. Regarding treatment, 21 patients (37%) received definitive surgical treatment and postoperative concurrent chemoradiation, 12 patients (21%) received surgery alone, 12 (21%) received chemoradiotherapy alone, 4 patients (7%) underwent surgery and received postoperative radiotherapy and 2  patients (4%) received either chemoradiotherapy or radiotherapy. Immunotherapy with anti-PD-1 was administered to 9 patients (16%).

**Table 1.** Relationships between anatomic site and clinicopathologic characteristics in patients with head and neck squamous cell carcinoma.

	Patients, n (%)	Oropharynx, n (%)	Oral cavity, n (%)	Larynx, n (%)	Hypopharynx, n (%)	p-value
Characteristics	57 (100%)	27 (47%)	15 (26%)	11 (19%)	4 (7%)	
**Age (years)**						
≤60	18 (32%)	7 (39%)	7 (39%)	1 (6%)	3 (17%)	0.046
>60	39 (68%)	20 (51%)	8 (21%)	10 (26%)	1 (3%)	
**Sex**						
Male	46 (81%)	22 (48%)	11 (24%)	11 (24%)	2 (4%)	0.11
Female	11 (19%)	5 (45%)	4 (36%)	0	2 (18%)	
**Smoking status**						
Yes	37 (65%)	19 (51%)	7 (19%)	9 (24%)	2 (5%)	0.22
No	20 (35%)	8 (40%)	8 (40%)	2 (10%)	2 (10%)	
**Alcohol status**						
Yes	5 (9%)	3 (60%)	1 (20%)	1 (20%)	0	0.88
No	52 (91%)	24 (46%)	14 (27%)	10 (19%)	4 (8%)	
**Differentiation**						
Basaloid	27 (47%)	24 (89%)	1 (4%)	1 (4%)	1 (4%)	**<0.0001**
Not Basaloid	30 (53%)	3 (10%)	14 (47%)	10 (33)%)	3 (10%)	
**p16**						
p16 positive	24 (42%)	19 (80%)	2 (8%)	2 (8%)	1 (4%)	**0.0003**
p16 negative	33 (58%)	8 (24%)	13 (39%)	9 (27%)	3 (9%)	
**PD-L1**						
<1%	9 (16%)	2 (7%)	3 (20%)	3 (27%)	1 (25%)	**0.001**
1–10%	20 (35%)	7 (26%)	5 (33%)	8 (73%)	0	
>10%	28 (49%)	18 (67%)	7 (47%)	0	3 (75%)	
**T stage**						
T1 or T2	20 (35%)	10 (50%)	7 (35%)	3 (15%)	0	0.184
T3 or T4	13 (23%)	3 (23%)	5 (38%)	3 (23%)	2 (15%)	
Not applicable	24 (42%)					
**Treatment**						
CRT	12 (21%)	9 (75%)	0	0	3 (25%)	**<0.0001**
Surgery	12 (21%)	1 (8%)	3 (25%)	7 (58%)	1 (8%)	
CTX or RT	2 (4%)	1 (50%)	0	1 (50%)	0	
Surgery and CRT	21 (37%)	11 (52%)	9 (43%)	1 (5%)	0	
Surgery and radiation	4 (7%)	1 (25%)	3 (75%)	0	0	
Lost to follow-up	6 (10%)					
**Immunotherapy**						
Yes	9 (16%)	6 (67%)	2 (22%)	0	1 (11%)	0.297
No	42 (74%)	17 (40%)	13 (31%)	9 (21%)	3 (7%)	
Lost to follow up	6 (10%)					
**Follow up**						
Alive	41 (69.0%)	19 (46.0 %)	11 (27.0 %)	8 (20.0 %)	3 (7.0%)	0.836
Deceased	11 (19.0 %)	5 (45.0 %)	4 (36.0 %)	1 (9.0 %)	1 (9.0 %)	
Lost to follow up	6 (10 %)					

Significant p-value is < 0.05 (bold).

CRT: Chemoradiation therapy; CTX: Chemotherapy; RT: Radiation therapy.

#### Histologic differentiation & correlation with anatomical site.racteristics

Fisher exact test was used. The null hypothesis was that clinicopathologic characteristics are independent and are not influenced by the anatomical site. We observed basaloid morphology in 27 (47%) of total cases (27/57). The majority of basaloid SCC were oropharyngeal tumors (24/27). In contrast, oral cavity, larynx and hypopharynx sites had low percentages of p16-positive patients 7% (1/15 patients), 9% (1/11 patients) and 25% (1/4 patients), respectively. In concurrence with others [13,14], we observed heuristically that basaloid differentiation in squamous cell carcinoma dominates in the oropharynx (p < 0.0001). In contrast, oral cavity, larynx, and hypopharynx had a conventional SCC (Table 1).

#### p16 expression & correlation with anatomical site

Positive p16 expression was detected in 24 (42%) patients and negative p16 expression was detected in 33 (58%) patients. Similar to basaloid differentiation, there was a striking predominance of high p16 expression in oropharyngeal (tonsil and base of tongue) SCC (19/27). On the other hand, oral cavity, larynx, and hypopharynx had low percentages of p16-positive patients 13% (2/15 patients), 18% (2/11 patients) and 25% (1/4 patients), respectively. In concurrence with others, we observed that p16 expression is more common in oropharyngeal (tonsil and base of tongue) squamous cell carcinoma (p = 0.0003) (Table 2A) [13,15].

**Table 2.** Correlation between p16, PD-L1 expression and basaloid differentiation of HNSCC.

A. Significant difference between p16 and tumor differentiation in patients with head and neck squamous cell carcinoma.					
Differentiation					
	Patients (n)	Basaloid	Not Basaloid	p-value	
p16					
Positive	24 (42%)	20 (83%)	4 (17%)	<0.0001	
Negative	33 (58%)	7 (21%)	26 (79%)		

B. Significant difference between p16 and PD-L1 expression (complete positive score) in patients with head and neck squamous cell carcinoma.					
PD-L1 (Complete positive score)					
	Patients (n)	<1%	1–10%	>10%	p-value
p16					
Positive	24 (42%)	1 (4%)	5 (21%)	18 (75%)	0.0027
Negative	33 (58%)	8 (24%)	15 (45%)	10 (30%)	

C. No significant difference between PD-L1 expression (complete positive score) and tumor differentiation in patients with head and neck squamous cell carcinoma.					
PD-L1 (complete positive score)					
	Patients (n)	<1%	1–10%	>10%	p-value
Differentiation					
Basaloid	27 (47%)	3 (11%)	7 (26%)	17 (63%)	0.167
Not Basaloid	30 (53%)	6 (20%)	13 (43%)	11 (37%)	

#### PD-L1 expression & correlation with anatomical site

**Figure 1.**
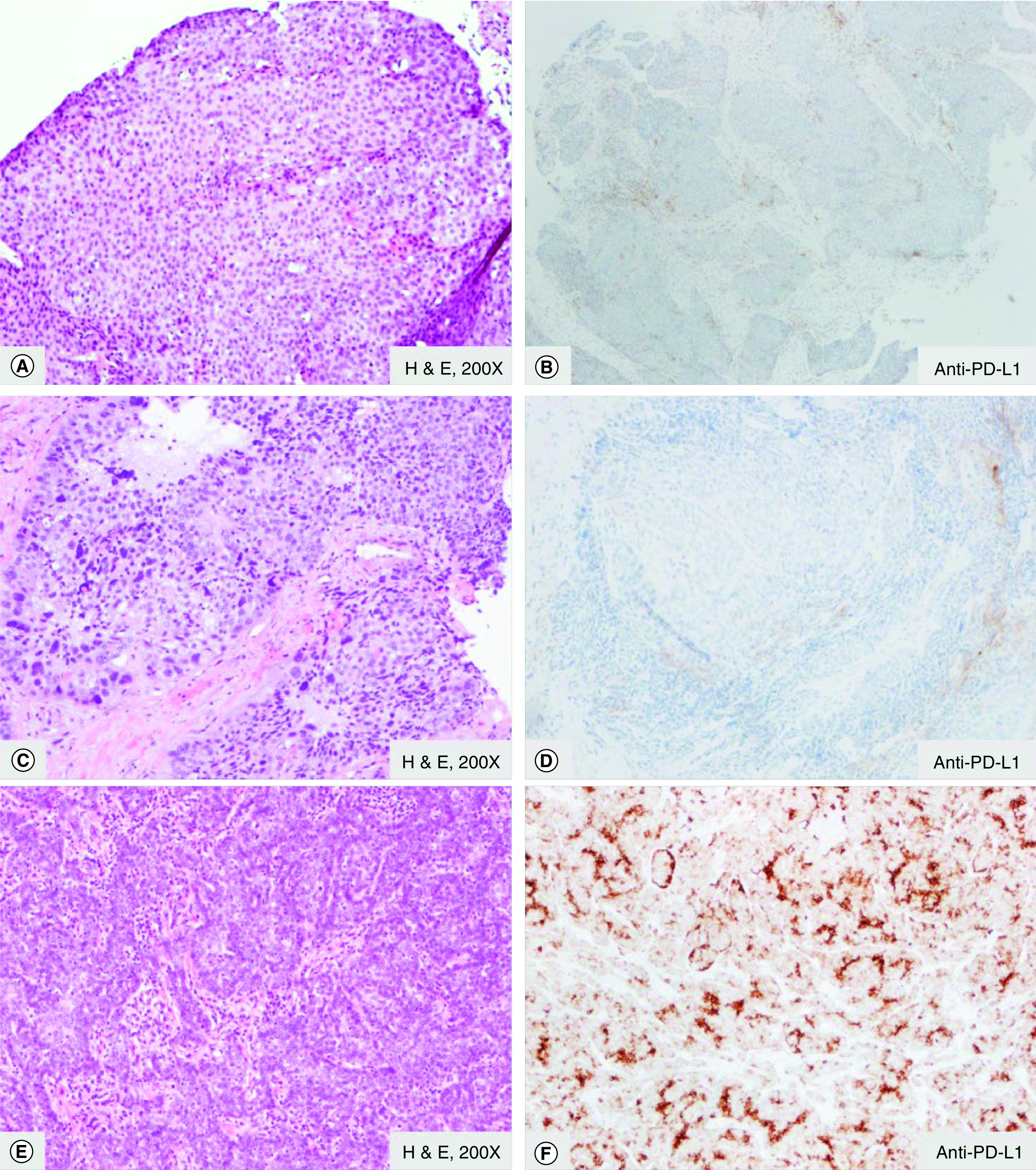
Squamous cell carcinoma of the head and neck shows different level of PD-L1 expression. **(A, C & E)** Squamous cell carcinoma. **(B)** Negative or <1% expression of PD-L1 (anti-PD-L1 SP263). **(D)** Low or 1–10% expression of PD-L1 (anti-PD-L1 SP263). **(F)** High >10% expression of PD-L1 (anti-PD-L1 SP263). H & E: Hematoxylin and eosin stain; PD-L1: Programmed cell death 1-ligand 1.

Immunohistochemical (IHC) analysis of p16 or PD-L1 expression was performed. CPS of low/absent (≤1%), intermediate (1–10%) and high (≥10%) was calculated in at least 200 cells to include tumor cells and mononuclear inflammatory cells (lymphocytes and macrophages) with any membranous staining. Representative staining patterns of PD-L1 in squamous cell carcinoma of the head and neck are demonstrated in Figure 1.

**Figure 2.**
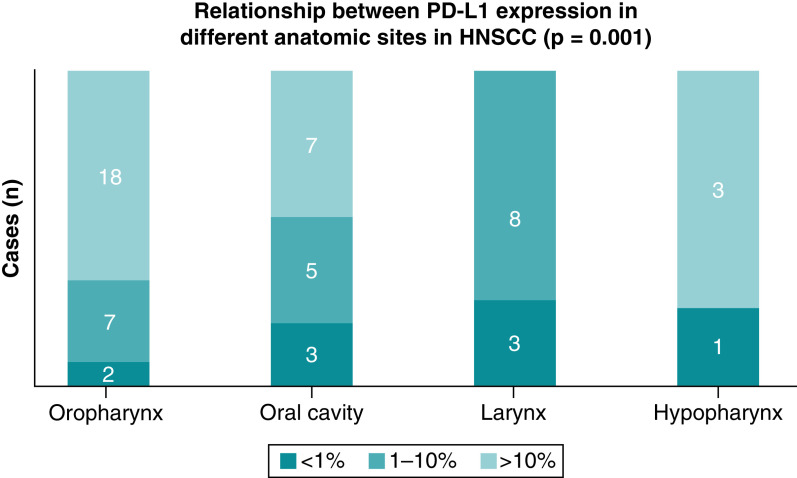
Relationships between programmed cell death 1-ligand 1 expression and anatomic site in patients with head and neck squamous cell carcinoma. PD-L1 (complete positive score), membranous staining on tumor cells and mononuclear inflammatory cells. Significant p-value is < 0.05. HNSCC: Head and neck squamous cell carcinoma; PD-L1: Programmed cell death 1-ligand 1.

PD-L1 CPS scores were high (≥10%) in 67% of the oropharyngeal SCC (18/27), 47% of the oral cavity (7/15), 75% of hypopharyngeal SCC (3/4) and 85% of the oral cavity SCC (12/14). Laryngeal SCC did not have high PD-L1 staining (0/11). The majority of laryngeal SCC cases had intermediate (1–10%) PD-L1 expression 73% (8/11 patients). In concurrence with others [16], we observed high levels of expression of PD-L1 in oropharyngeal (tonsil and base of tongue) squamous cell carcinoma (p = 0.0014) (Figure 2, graphic representation).

**Figure 3.**
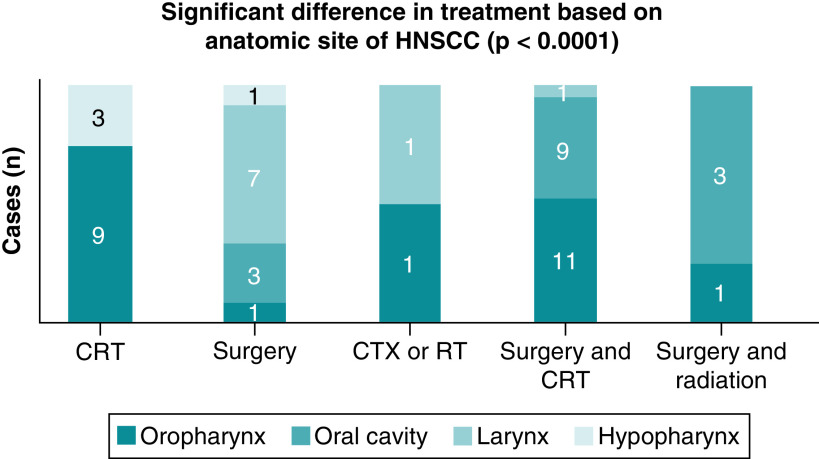
Relationships between anatomic site and treatment modalities in patients with head and neck squamous cell carcinoma. Significant p-value is < 0.05. CRT: Chemoradiation therapy; CTX: Chemotherapy; RT: Radiation therapy.

Treatment modality is primarily guided by the anatomical site of the HNSCC [17]. There was a significant difference in the treatment modality that patients received in relationship to the site of involvement by HNSCC (p < 0.0001) (Figure 3, graphic representation) [17].

### Correlation between p16, PD-L1 expression and basaloid differentiation of HNSCC

The relationships between p16 expression, basaloid differentiation and PD-L1 expression are presented in Table 2A & B, respectively. We observed that basaloid differentiation in HNSCC was more common in association with p16 expression (p < 0.0001). PD-L1 expression was higher in p16-positive HNSCC (p = 0.0027) (Table 2B). Although we noted a trend to the increased PD-L1 expression in HNSCC with basaloid histology, there were no significant differences between basaloid differentiation and PD-L1 expression (Table 2C).

## Discussion

We investigated associations between expression of p16, PD-L1 and basaloid morphology in 57 patients with HNSCC. All cases of HNSCC were associated with the mucosa to include 27 oropharynx, 15 oral cavity, 11 larynx and 4 hypopharynx cases.

Basaloid morphology in squamous cell carcinoma is considered to be an aggressive variant of squamous cell carcinoma [10,13]. In contrast, OPSCC commonly shows basaloid features, but overall survival remains superior to conventional HNSCC [18]. Therefore, histologic grading of OPSCC remains to be a limited prognostic value. We corroborated previous reports on the predominance of the basaloid morphology of HNSCC in oropharynx [13]. We also report that laryngeal SCC does not commonly have basaloid differentiation.

In concurrence with previous studies, we found strong p16 expression in oropharyngeal SCC (p = 0.0003) [2]. p16 expression correlated with prognosis as demonstrated by previous work [19]. p16 expression was negative in 9 out of 11 patients with oral squamous cell carcinoma. We did not find a significant difference in survival with p16 positivity (p = 0.74). According to the published data, p16 was not an independent predictor survival in patients with oral squamous cell carcinoma [20]. Hypopharyngeal squamous cell carcinoma did not show an increase in p16 expression and was not a good predictive biomarker for the survival [21]. In our study, 9 out of 11 patients with laryngeal carcinoma demonstrated negative p16 status by immunohistochemical stain. In previous reports laryngeal carcinoma showed higher proportion of p16-positivity, but p16 was not prognostically significant in predicting survival [22]. In contrast, oral cavity, larynx and hypopharynx SCC did not show an increase in p16 staining.

In regard to PD-L1 expression, we noted that 85% of all of the cases demonstrated intermediate or high PD-L1 expression (50/57 cases). In concurrence with previous studies, we observed that 67% of oropharyngeal SCC have high levels of PD-L1 expression [16,23]. We did not find a significant difference in survival with levels of expression of PD-L1 (p = 0.63). Interestingly, previously published results suggested that high PD-L1 expression might be a biomarker of an adverse prognosis in patients with oral squamous cell cancer [24]. Our analysis demonstrated that PD-L1 expression was high in hypopharyngeal carcinoma. There are data to suggest that overexpression of PD-L1 can act as a significant biomarker for the adverse clinicopathologic features and poor prognosis of patients with hypopharyngeal squamous cell carcinoma [25]. Our study reported that PD-L1 expression was high in three out of four patients with laryngeal squamous cell carcinoma. There is no previously reported study to suggest a relationship between PD-L1 expression and survival in patients with laryngeal squamous cell carcinoma [26]. Further studies to correlate PD-L1 and p16 expression with survival are necessary.

Our study has several limitations. It is a heterogeneous retrospective cohort involving a small number of patients. Further studies to include a larger patient sample size are necessary. Evaluation of complete positive score of PD-L1 expression by immunohistochemistry is subjective.

In conclusion, we demonstrated significant relationships between p16, PD-L1 expression, and basaloid differentiation in HNSCC.

## Future perspective

Further studies, especially prospective, in a larger cohort are necessary to correlate PD-L1 and p16 expression with survival. It is important to study larger number of squamous cell carcinoma involving hypopharynx, larynx, and oral cavity to elucidate clinical significance of the histomorphology, p16, and PD-L1 expression.

**Summary points**

**Background**

- Squamous cell carcinoma is the most common malignancy in the head and neck area.
- Oropharyngeal squamous cell carcinoma is the most common type of squamous cell carcinoma.
- Human papilloma virus (HPV)-associated oropharyngeal squamous cell carcinoma has a superior outcome.
- P16 is a surrogate marker for HPV infection.
- Programmed cell death 1-ligand 1 (PD-L1) is a powerful biomarker that can be useful to justify immunotherapy with anti-PD-L1 (Keytruda).

**Materials & methods**

- Retrospective analysis of 57 cases was performed.
- Fisher exact test was used to calculate p-values.
- Study approved by the Institutional Review Board (IRB) of University of California, Irvine Medical Center (HS-2019-5255).
- Immunohistochemical staining was used to assess p16 and PD-L1.
- Immunotherapy with anti-programmed cell death protein 1 (anti-PD-1) was administered to 9 (16%) patients.

**Results**

- There were 46 men (81%) and 11 women (19%).
- Oropharyngeal SCC constituted 47% of all cases.
- Basaloid morphology was observed in 47% of cases. The majority of basaloid SCC were oropharyngeal tumors.
- Basaloid differentiation was more common in association with p16 expression.
- p16 expression was high predominantly in oropharyngeal SCC (19/27).
- PD-L1 expression was high (≥10%) in 67% of the oropharyngeal SCC (18/27).
- PD-L1 expression was higher in p16-positive SCC.
- Treatment modality depended on the site of involvement by SCC.

**Discussion**

- Although basaloid morphology is associated with aggressive SCC, oropharyngeal SCC has a superior prognosis.
- Grading of oropharyngeal SCC is of limited value.
- There is no correlation between p16 expression and survival.
- Oropharyngeal SCC has high levels of PD-L1.
- PD-L1 expression did not offer prognostic significance.

**Further perspective**

- Studies with a large cohort are necessary to draw conclusion about survival.
